# Hope and despair: a qualitative exploration of the experiences and impact of trial processes in a rehabilitation trial

**DOI:** 10.1186/s13063-019-3633-8

**Published:** 2019-08-23

**Authors:** Meriel Norris, Leon Poltawski, Raff Calitri, Anthony I. Shepherd, Sarah G. Dean, on behalf of the ReTrain Team

**Affiliations:** 1 College of Health and Life Sciences, Brunel University London, Uxbridge, UB8 3PH UK; 2 University of Exeter Medical School, Exeter, EX1 2LU UK; 3 Department of Sport and Exercise Science, University of Portsmouth, Portsmouth, PO1 2ER UK

**Keywords:** Trial design, Randomisation, Equipoise, Qualitative, Stroke, Rehabilitation

## Abstract

**Background:**

Unanticipated responses by research participants can influence randomised controlled trials (RCTs) in multiple ways, many of which are poorly understood. This study used qualitative interviews as part of an embedded process evaluation to explore the impact participants may have on the study, but also unintended impacts the study may have on them.

**Aim:**

The aim of the study was to explore participants’ experiences and the impact of trial involvement in a pilot RCT in order to inform the designing and delivery of a definitive RCT.

**Methods:**

In-depth interviews with 20 participants (10 in the intervention and 10 in the control group) enrolled in a stroke rehabilitation pilot trial. A modified framework approach was used to analyse transcripts.

**Results:**

Participation in the study was motivated partly by a desperation to receive further rehabilitation after discharge. Responses to allocation to the control group included an increased commitment to self-treatment, and negative psychological consequences were also described. Accounts of participants in both control and intervention groups challenge the presumption that they were neutral, or in equipoise, regarding group allocation prior to consenting to randomisation.

**Conclusions:**

Considering and exploring participant and participation effects, particularly in the control group, highlights numerous issues in the interpretation of trial studies, as well as the in ethics of RCTs more generally. While suggestions for a definitive trial design are given, further research is required to investigate the significant implications these findings may have for trial design, monitoring and funding.

**Trial registration:**

ClinicalTrials.gov, NCT02429180. Registered on 29 April/2015.

## Background

Trial integrity is a key concern for clinical research to ensure as much as possible that protocols are followed and validity is enhanced. Randomised controlled trials (RCTs) epitomise the drive to reduce bias and promote internal validity in the hope that outcomes can be attributed to the intervention components. Despite ongoing debate, RCTs (and systematic reviews of RCTs) are still considered the pinnacle of primary clinical research [1], and the inclusion of process evaluation assists in the ability to monitor and record the integrity of a complex trial as is typical in rehabilitation research [2, 3]. Specifically, qualitative data collection facilitates exploration of experiences and factors not previously considered or known, which could impact on the trial and can therefore inform trial development. However, process evaluation also potentially exposes contextual factors, including research participation effects [4]. Within unblinded clinical trials, as is common in rehabilitation, these effects may include compensatory rivalry and resentful demoralisation, forms of performance bias usually associated with control groups. The former refers to an increase in performance in order to compete with the intervention group, the latter a disengagement from performance due to frustration with allocation. Both have been recorded in trials but are considered poorly understood and inconsistent in presentation [5]. Patient preference to allocation has also been shown to influence trial outcomes, although clarity on the extent of this influence is variable [6, 7].

Reasons for choosing to participate have also been explored, with the suggestion that those volunteers less driven by altruism may demonstrate stronger personal influences on the study, for example, by compensatory rivalry [4]. Other concerns include misunderstanding of the trial processes, which can result in “therapeutic misconception” where the research process is conflated with therapeutic intentions [8]. Such individual interpretations of formal research processes and documentation may result in behaviour change, but also fundamentally challenge concepts of informed consent and the ethical positioning of clinical trials [9]. The World Medical Association [10] through the Declaration of Helsinki requires that consent be based on appropriate information regarding potential risks and benefits or participation, and this would include the impact of trial processes such as allocation. Some researchers have further suggested that potential participants must also accept the process of randomisation and do so from a position of equipoise [11]. The latter point refers to an informed acceptance and lack of preference for allocation to the intervention or control group [12]. Indeed, Wade et al. ([13]:2025) suggests that “randomisation is only ethical where evidence of equipoise emerges”. However, research suggests that such informed equipoise is often lacking in participants and the recruiting researchers [14–17].

McCambridge et al. [5] suggest that the there is a need for more empirical work in order to elaborate the details of research participation effects, and this paper is a response to that call. Its aim is to explore the experience and impact of involvement in a pilot RCT with a focus on trial processes (e.g. recruitment, randomisation and communication) rather than the intervention itself. Specific objectives are to explore the reasons for participation and the perceived impact of allocation on the individual, with a view to informing the design and delivery of a definitive RCT.

## Method

This qualitative study was embedded within an external pilot RCT with parallel process evaluation [18, 19]. An external pilot RCT is not embedded in a full RCT (unlike an internal RCT) and aims to test the main components in preparation for a full RCT. The trial assessed the feasibility and acceptability of a 12-week functional training programme for community-dwelling adults with stroke. All participants had been discharged from National Health Service (NHS) physical rehabilitation programmes prior to starting the trial. Group allocation was randomised using a computer-generated algorithm, which considered time since stroke and functional disability [19]. The control group within the trial received treatment as usual and an advice booklet about exercise after stroke [20]. Treatment as usual varied between individuals and while we requested that they did not participate in formal physical rehabilitation, we did not prevent them from doing so. Whether they did participate in other treatments was not formally assessed. The intervention group received twice weekly group training on functional rehabilitation, including task-related practice, targeted strength training and adaptations to tasks such as getting on and off the floor. These were based on the action for rehabilitation from neurological injury (ARNI) principles [21] and delivered by ARNI-trained fitness professionals. This was complemented with additional targeted homework and goal setting to enhance self-management. The main outcomes focused on feasibility, fidelity and acceptability. Functional outcomes included a suite of measures primarily Rivermead Mobility Index, Timed Up and Go, modified Patient-Specific Functional Scale and objective physical activity monitoring through wrist-worn accelerometers, supplemented with a diary. A range of other secondary outcomes were also included [19]. The process evaluation was designed to examine fidelity to the defined programme in addition to feasibility and acceptability as recommended for complex rehabilitation interventions [3]. As part of the process evaluation a purposive sample of participants in both the intervention group and the control group were interviewed at the end of the intervention period of the trial (approximately 6–7 months post randomisation) in order to ascertain their experiences of the trial in general, including the trial processes. The data in this paper are drawn from these interviews, which explored both specific experiences of trial involvement and perceived response to the intervention (considered in detail a separate paper [22]).

A qualitative approach to this aspect of the process evaluation was taken because of the necessary focus on the participants’ experience [23]. Methodologically the study drew on constructionist and phenomenological traditions in order to represent the influence of the context of the training group structure while remaining focused on the experiences of the individuals themselves (see [22] for further details). A sampling frame was created based on characteristics potentially influencing process and outcome, including gender (male/female), age (younger, ≤ 60 years/older, 61+ years), cohort (1a, 2 or 3), level of disability post stroke (as measured by the modified Rankin Scale (MRS) 0–2, mild/3–4, moderate), self-reported exercise proclivities pre stroke (exerciser/non-exerciser), time since stroke (more or less than 12 months) and, for the intervention group, participation level in training (high or low based on attendance). The pilot trial had 45 participants randomised between the intervention and control groups, and 10 in each group were invited and subsequently consented to interview. This number was selected as it met the needs of the sampling frame (i.e. all criteria were represented), was feasible and avoided unnecessary participant burden.

The trial ran in four cohorts relating to different locations and recruitment periods. All individuals who at the point of consent to the main trial had agreed to be contacted to consider taking part in an interview were entered in the sampling frame (43 participants). Ten participants each from the control and intervention groups were purposefully sampled, ensuring that all sampling criteria were met. The trial manager (RC) contacted potential interviewees and invited them to interview. On agreeing to participate, contact details were passed onto the interviewer (LP), who arranged the visit.

Semi-structured one-to-one interviews were conducted in the location of participants’ choice (their home for all) by an experienced qualitative researcher. The researcher was associated with the trial and therefore had knowledge of both the trial and intervention but was not involved with recruitment, intervention delivery or assessment. The interviews followed topic guides, which had been previously developed through reference to the literature and discussions within the research team including representation from stroke survivors. This paper specifically focuses on questions that related to study involvement rather than experience of the intervention itself (see Table 1 for example questions).

**Table 1:** Indicative interview questions

Example question	
Why did you volunteer to take part in this study?	
Did you have any concerns or fears about taking part?	
What did you think was involved in the programme before you took part?	
What did you think about the fact that you could have been put into either the group receiving supervised training or the group that received an exercise leaflet?	

**Fig. 1:**
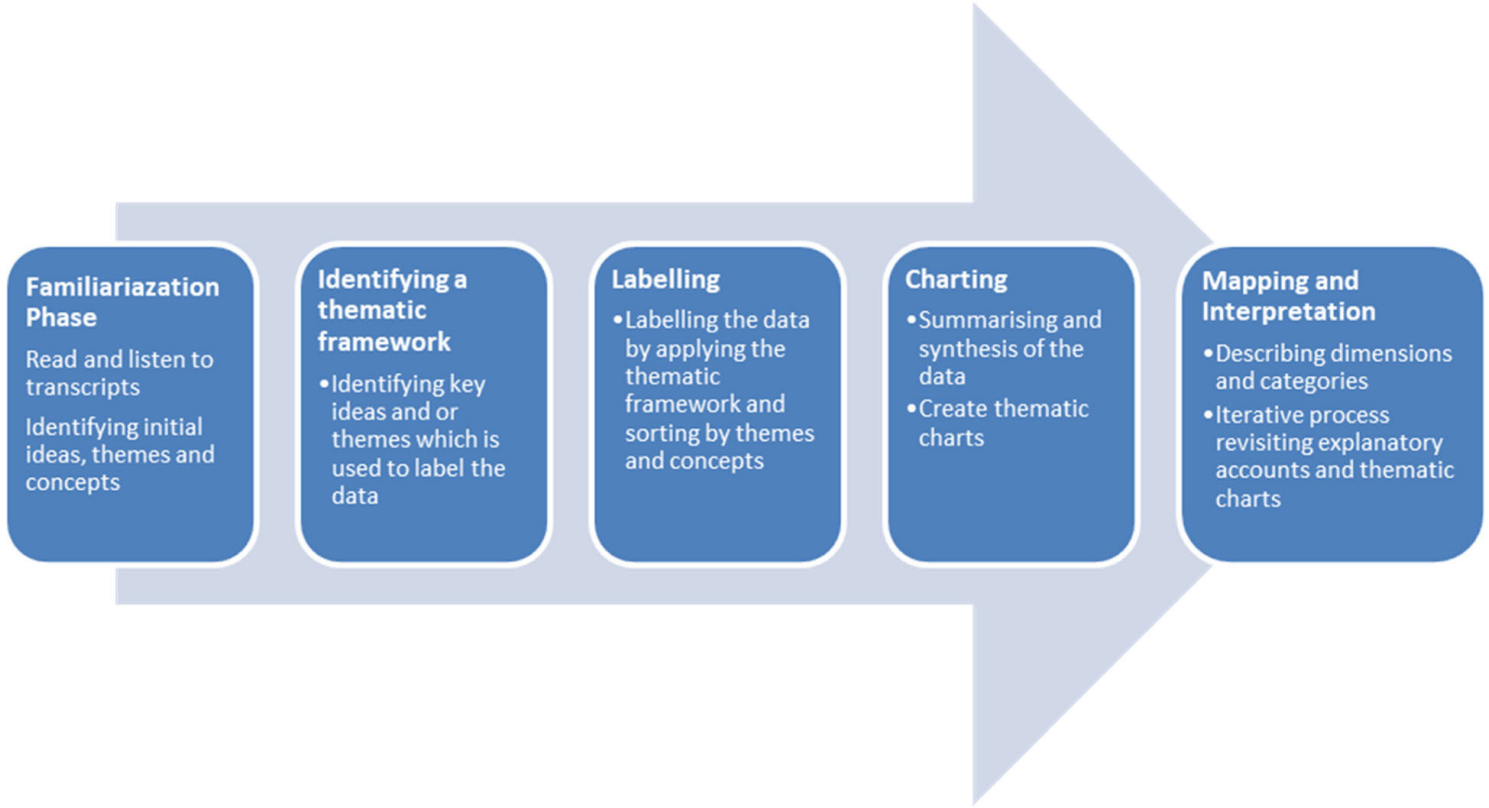
Phases in Framework Analysis [24]

All interviews were audio-recorded and transcribed verbatim. The transcripts were analysed by an experienced qualitative researcher (MN) who was aware of the content of the intervention but not involved in its delivery or assessment. A framework analysis [24] was undertaken to explore the data using both the deductive and inductive approaches. Deductive codes were applied to aspects that were specifically asked about in the topic guide, e.g. acceptability of communication with the research team; however, the majority of codes and all themes were inductive. This involved several phases as illustrated in Fig. 1.

Additional negative case analysis, in which reference to views opposed to emerging themes are specifically considered, was undertaken between the labelling and charting phases. The initial analysis was completed independently for each group of participants (intervention and control) but combined at the labelling phase for aspects relating to trial involvement. All data collection and analysis were completed blind to the results of the study itself, with additional information about the quantitative results of the study added later.

Discussions were held with the other members of the trial team (SD/RC/LP) during analysis and writing. These discussions were not deemed to be a validation process, but rather an opportunity for critical reflection on the depth of the analysis and appropriateness of interpretations drawn [25].

Ethical review was conducted and approved by NRES Committee South West – Cornwall & Plymouth (REC ref. 15/SW/04) and participants were given further information and provided further written consent for the interview phase of the study.

## Results

Characteristics of the interviewees are given in Table 2. These include 10 participants from the intervention group (6 male, mean 44 months since stroke - range 2–120 months, MRS 2–3, mean age 72 years - range 56–91 years) and 10 from the control group (6 male, mean 65 months since stroke - range 5–204 months since stroke, MRS 1–3, mean age 72 years - range 48–89 years). This is representative of the participants in general and of a chronic community-dwelling population of stroke survivors, and met all our sampling frame criteria. The interviews lasted on average 42 min (range 12–78 min).

**Table 2:** Participant characteristics

Trial arm	Participant number	Cohort	Time since stroke (months)	MRS*	Age	Sex	Programme attendance categorisation	Pre stroke exercise tendencies**
Control	2	1a	36	2	77	F	n/a	Exerciser
Control	3	1a	40	3	81	M	n/a	Non-exerciser
Control	7	1a	5	3	73	M	n/a	Exerciser
Control	8	2	54	2	64	M	n/a	Exerciser
Control	12	2	11	3	89	M	n/a	Non-exerciser
Control	14	2	53	3	48	M	n/a	Non-Exerciser
Control	19	2	30	2	63	F	n/a	Non-exerciser
Control	35	3	204	2	82	F	n/a	Exerciser
Control	39	3	96	2	70	F	n/a	Non-exerciser
Control	41	3	120	1	74	M	n/a	Non-exerciser
Intervention	4	1a	36	3	64	M	High	Non-exerciser
Intervention	5	2	4	3	73	M	Low	Non-exerciser
Intervention	6	1a	42	3	91	M	High	Non-exerciser
Intervention	9	2	18	3	56	F	High	Exerciser
Intervention	16	1a	2	3	89	M	High	Non-exerciser
Intervention	22	2	120	2	77	M	Low	Exerciser
Intervention	25	2	33	3	80	F	High	Non-exerciser
Intervention	30	2	13	2	59	M	High	Exerciser
Intervention	32	3	84	2	67	F	Low	Non-exerciser
intervention	43	3	90	2	68	F	Low	Exerciser

*MRS = Modified Ranking Scale (measuring level of disability - higher value indicates greater disability)

**Attendance categorisation: the amount of intervention received and engaged with by the participant, dichotomised into high (>60% attendance) versus low (<60% attendance)

This section presents the two main themes derived from the data: the subsection “An offer too good to refuse” explores the reasons participants gave for volunteering for the study. The combined drivers of gratitude, desperation, and acceptance of professional guidance highlight particular ethical concerns. The subsection “The nature of control” examines the responses to group allocation and how participants experienced participation in the research processes. Here, primacy is given to the experience of control-group allocation, due to the strength and diversity of their responses and scarcity of literature in this area. In line with the qualitative approach, interpretation of the participants’ words is presented alongside the quotations. In the presentation of the themes, study ID is used for supporting quotations with basic demographic descriptors (gender_ age), group allocation and transcript line number.

### An offer to good to refuse

Participants expressed multiple motivations for their involvement in their study, resulting in two sub-themes; Doing it because of others, and “I would have done anything”. Combined, they suggest influences that challenge concepts of equipoise in decision-making when volunteering to take part in a RCT.

#### Doing it because of others

It was evident in the transcripts that a number of the participants joined the study due to past or ongoing experiences and advice. For example, one was motivated by the recommendation of their general practitioner (GP) with whom they had a long and trusted relationship:

I had a letter from the GP … so she said ‘I wonder if you would like to, you know go for it?’ … I thought ‘right well she sent me the letter so she must think well there could be some good to come out of it. (32,F/82 CG:205–209, 262).

Another suggested that the fact the study was funded by the Stroke Association gave it a credibility that they were willing to trust. A more general influence was previous care they had received in their stroke journey, as participant 6 describes:

I wanted to say thank you for all the help I had been given … everybody has been so kind and helpful (6:M/91 IG:47, 78).

The participant continues with a description of the lack of expectation he had for his own personal benefit, describing participation as an act of giving and contribution. In this case the gratitude was directed primarily at the individuals who had introduced him to the study and not his formal NHS care.

While past kindnesses were referred to as a key motivation, future projections were also inferred. Participants demonstrated an understanding that the research endeavour was an investment in future therapeutic benefits, mainly for other people. Being part of the process of developing a better future for other stroke survivors was an important element in their decision-making and something they found rewarding:

I think that I wanted to help, because I had the stroke and I wanted to be part of the team that could help. It was a new thing that they were starting, a new study and I am a bit … I thought that if I can help in these situations, you know I am willing and I am up for it. (30: M/59 IG:124–127).

I felt it would only benefit other people … having done research for the medical school in the hope, helping other people ... And I thought well if it helped other people with stroke then I would go for it (7: M/73 CG:283–289).

This reference to research and benefit to others rather than yourself was only directly expressed by those in the control group (participants 3, 7, 30, 35, 39). This may suggest that its emphasis is heightened retrospectively in the absence of their own individual therapeutic benefit.

A third aspect of this sub-theme was the process of participation selection itself. One participant described how being selected and clearing the screening process demonstrated that they had been “chosen”. As such that selection endowed a gratitude that they intended to honour through participation in the study:

I had a clear mind. I thought ‘right go for it, whatever they, I have been offered’. I am very grateful and that I have been chosen to take part. (43: F/68 IG 151–152).

This last aspect was unsurprisingly from a participant in the intervention group. The potential implications of not “being chosen” are discussed later.

#### “I would have done anything”

In contrast to the altruistic position described above was one that was firmly situated around personal drivers and the expectation that inclusion would impact positively on *their* life. This was the most common reason for participation, although most strongly articulated by participants in the intervention group:

I just want to do anything. Anything to get better; to better myself … if someone came along tomorrow and said there’s a new exercise on the moon, I would be the first one there. (4: M/64 IG:91–93).

Because I would have done anything … to be able to get back to a bit of normal life again you know. Because this is not my life sitting like this no way. (25: F/80 IG:229–231).

Within these narratives was a strong sense of desperation for help, a willingness to try anything. While participant 25 (F/80 IG) suggests that this motivation/desperation is driven by a desire to change a current status that they consider incompatible with their description of life, others looked to external factors. For example, participant 30 (M/59 IG) discussed a conversation he had with other participants, which reflected on their previous rehabilitation care:

When I left the physio, I thought is that it now … no further to continue and actually it was one of the main points that many people brought up. One of the guys said ‘it’s as if we are now on the scrap heap, that’s the end of it’ (30: M/59 IG:191–198).

It would appear from these narratives as if the combination of dissatisfaction with their current status and the sense that they had been neglected or deserted by their formal rehabilitation guides left the participants desperately searching for other opportunities.

Linked with this was the expressed view that some improvement was expected as a result of inclusion in the study. For most this was a vague expectation that it might “make a difference” (19: F/63 CG:270) or “I thought it may help me” (25: F/80 IG:229). For others, more specific expectations were expressed. For example, participant 41 (M/74 CG) said he did not think the exercises he was currently doing were appropriate or the most beneficial and he hoped that this intervention might give him the “right sort of exercises”. Participant 6 (M/91 IG) in contrast was persuaded that the cardiovascular benefits were an important factor and were key to his decision to participate.

Overall this theme suggests complex and various influences on the motivation to participate in the study. Differences between the control and intervention group are noted, particularly in relation to doing it because of others or expected personal gain, respectively.

### The nature of control

This theme explores the participants’ responses to their allocation and their experiences of the trial processes. It is perhaps unsurprising that the narratives from the control group contained a richer engagement with this line of enquiry, and offer an array of important insights into the experience of being the “ones who lost out” and critically the impact of that perceived “de-selection” on the behaviour of individuals. This section presents two sub-themes in the subsections “Acceptance but potentially cast adrift”, through which the necessity of research, personal circumstance and contact in the research are explored, and “Determination to change”, where the allocation process itself becomes an active part of an intervention.

#### Acceptance but potentially cast adrift

Allocation was met with positivity in the intervention group. Participants wanted to “open their eyes to something new” (4: M/64 IG:1193) and “be chosen to do the proper thing” (6: M/91 IG:905), and this gave them the opportunity to achieve that:

Well I was a bit worried I wasn’t going to get into it you know. I thought I was you know, I’m going to miss out … but I didn’t so I got the right one (5: M/73 IG:814–817).

All indicated that allocation to the control group would have disappointed them, with four participants (4,5,22,43) suggesting they may have dropped out of the trial as there would be little personal advantage with continuing. For six of the participants in the control group, their allocation was not a significant concern. They had understood the control trial process and the requirement for some people to end up not having the intervention. Participant 35 describes the acceptance of this allocation clearly:

I accepted that you can’t really do something like this without actually having a control group can you? ... I was interested in what I could get out of it, but it didn’t bother me terribly that I didn’t get in. (35:F/82 CG:312–321).

Not all of the participants were as clear about the role and need of the control group, but nevertheless they accepted their allocation. In some cases, such as participants 12 and 35, they were somewhat relieved as they either perceived exercise to not really be their thing or were concerned by the burden of the twice weekly class obligation. Such narratives hint at the possibility that allocation to the intervention group may have resulted in unwilling participants or poor attendance.

In contrast, four participants described quite a negative reaction to being in the control group and indeed one dropped out of the study, citing unhappiness in their allocation as the reason. Perhaps unsurprisingly, disappointment was the most commonly expressed emotion.

“Yeah, I was disappointed … I should think everybody was, wasn’t they?” (19: F/63 CG:439–443).

I appreciated that it was a fifty/fifty chance of help and I felt very guilty that I didn’t sort of get myself together to explore. But I did feel quite disappointed to be honest. (2:F/77 CG:165–167).

This quote from participant 2 hints at the implication of that disappointment. Her reference to guilt suggests a self-expectation that when she was recruited into the control group this should have prompted her to explore other opportunities for rehabilitation, an expectation she was unable to enact. This participant had a history of depression and her narrative continues to suggest that the fact that she was unable to change her physical condition contributed to a recent self-reported deterioration in her mental health and restarting of associated medication.

While not referring to depression, participant 19 describes a sense of being a little invisible as illustrated in this passage, which followed a discussion about whether the control group should be given more exercises to do.

Well it might have made me feel like I wasn’t just cast adrift … I think especially probably for the people in the control group because they’re not going out to classes and doing anything. To make them feel like they’re contribution is worth having. And that you’re not just abandoned (19: F/63 CG:993–1007).

This sense of abandonment and being “cast-adrift” is noted despite the fact that all participants commented that they were satisfied with the communication with the research team over the research period.

It is perhaps relevant to note that many of the control group commented on the practical irrelevance of the usual-care booklet that they were given. With the exception of participant 3, all were aware of the booklet in some form or other and most remembered reading it. However, the vast majority agreed that if they remembered the content at all, it was too generalised, covered ground they already knew and therefore did not impact on their activities.

I thought it would be more specific to stroke. I think I thought it would tell me something I didn’t already know maybe or just set me certain tasks that I could tick off. (2: F/77 CG:177–179).

Interviewer (Int): Do you remember getting that leaflet?

Participant (Part): Vaguely yes.

Int: Did you look at it?

Part: I must have done.

Int: Did it make any difference receiving that or reading it?

Part: Hmmm, I don’t think so. (12: M/89 CG: 524–534).

In contrast to this generally perceived lack of impact of the leaflet, a few participants did like it. Three participants (41, 14 and 8) all commented positively on the content in a general fashion. However, only one indicated that reading the leaflet made any difference to their actual activity.

The leaflet was given as part of usual care and was not designed to change behaviour particularly. As a result, the fact that it does not appear to have done so is not a major concern. However, its presence but lack of utility does have the potential to create frustration if expectations are inadvertently raised in this group of individuals who are desperate for their current condition to change.

#### Determination to change

This final sub-theme focuses on the actions taken by some participants in apparent response to their allocation to the control group. Despite the general disappointment of allocation to the control, most participants accepted their position and reported continuing activities as normal. However, for 4 of the 10 interviewees, allocation to the control group was a prompt for action. The nature of that prompt differed between participants. For participant 3, inclusion in the study became an indication that they were no longer invisible in a world that seemed not to notice her condition. As a result, this changed her response to her current situation.

It is nice to know that somebody cares … and it [the study] made a difference. Oh yes of course it did yeah … it gives you a little bit of hope. A bit of encouragement … I am determined to keep going (3: M/81 CG 298–327).

She goes on to discuss her commitment to the gym and also regularly completing exercises she had previously been given from the hospital. This sense of keeping going and action despite allocation to control was shared by participant 8. Participants 7 and 14, however, had a more active response to their allocation. Participant 7 immediately joined a gym and reported increasing their activity levels, and participant 14 and their carer were quite robust in their response.

Well bugger them; we are going to prove them that you don’t need expensive trainers … I would describe myself as slightly bloody minded and stubborn and if it doesn’t go my way you know I will do what I can to prove everyone wrong. (14 carer:1158–9/ 1181–1186).

It was like blooming heck, all this for … Hold on. Alright, come on we’ll go and get that done. (14: M/48 CG:1192–1193).

They set goals and described an active regime of specific exercises and more general physical activity in order to create their own rehabilitation programme. They later described some of the benefits both in movement and everyday life that they had perceived as a response.

Such narratives are essential to capture as they challenge the idea that the control group simply continue with usual care, and in this specific case, the narrative gives support to concepts of compensatory behaviour change. If the involvement in the study itself creates an environment of intervention, then the gap between control and intervention group is at best blurred and narrowed at worst, potentially undermining the purpose of a controlled trial.

## Discussion

This study aimed to explore the experience and the potential impact of participation in a stroke rehabilitation trial. In relation to the specific objectives, the reasons for participation were found to be complex, and in some cases influenced the individual’s trial experience. Group allocation was viewed in different ways by participants, but allocation to the control group may have negatively impacted some individuals, and may also have undermined control group fidelity in the study. More specific discussion and comparison with the literature is given below illuminating the participant effects in the trial. This is followed by recommendations, based on our findings to help inform the design and delivery of a definitive RCT.

The first issue raised through these interviews is the complexity of ethics within consent for this trial. While altruism was expressed as a motivator, which is consistent with other studies [26, 27] and was prominent in the narratives of the control group [16], it was not the only reason cited for participation. Expectations of direct personal benefit of inclusion coincided with apparent endorsement from key stakeholders, in this case the research funder and the clinicians who were involved in potential participant identification. This combination may lead to assumptions of therapeutic benefit of participation, which aligns with concerns of therapeutic misconception [8] and this has been discussed in other studies [5, 27]. These influences are important to consider as they demonstrate the potential power of these stakeholders regarding the project and the projection that they are also endorsing its content. It re-emphasises the importance of these figures being mindful of their role as gate-keepers and guides in the decisions potential participants may take. However, removing this endorsement could undermine recruitment rates and it may rather be for researchers, who are obtaining the consent, to be more vigilant in ensuring participant equipoise at the time of recruitment.

The apparent focus on the positive potential for participation links rather uncomfortably with the expressed desire or desperation for improvement. It is beholden on researchers to ensure that participants have considered the personal implications of taking part in the study in a balanced and informed fashion before consenting, although their capacity and desire to do so has been questioned for some time [8, 13]. However, it is less clear how this can be appropriately negotiated and ensured when potential participants are willing to try anything, particularly anything apparently endorsed by trusted sources. Furthermore, evidence suggests that participants do not fully engage with participation information sheets [16, 28], which implies the need for an even stronger emphasis on the ethical imperative for the recruiting researcher to establish equipoise in their personal interactions with potential participants [14, 29].

Ethically, participants in a trial such as this are described as vulnerable, but the nature of that vulnerability is rarely articulated in detail. These narratives suggest that an individual’s very desire/desperation to participate in a trial may render them vulnerable to suggestion, which in turn disrupts the equipoise deemed essential for ethical consent-gaining and hence for effective randomisation [10]. Such imbalances in decision-making parallel those seen in treatment decision-making in general healthcare [30, 31] and other clinical trials [16, 17], and create an ethical challenge for trial managers and recruitment personnel worthy of further enquiry.

A further ethical concern raised in these interviews was the potential implication of perceived rejection [16]. The narratives from the control group indicated a sense of being forgotten, or set aside as the “unchosen” despite their regular contact with the research team. It is particularly relevant that these narratives of abandonment mirror those of stroke survivors when describing their experience of rehabilitation more generally, creating a double loss [32, 33]. From an ethical perspective the most concerning was the potential implications for the mental health of participants, particularly pertinent given the high rates of depression in people post stroke [34]. Therapeutic expectations, heightened through recruitment but later thwarted through allocation to the control group, may exacerbate feelings of abandonment and hopelessness already experienced by some stroke survivors when their rehabilitation ends. It is possible to hypothesise that this impact was exacerbated by the use of a non-active control and other studies have reported participants feeling “swindled” when allocated to the control group [35]. However, studies using active controls have also illustrated concerns with treatment preference and acceptance of equipoise in the randomisation process [29].

All clinical trials have processes in place to record adverse events during the course of a trial. These accounts highlight the importance of monitoring, recording and (if appropriate) acting on psychological as well as physical events that may occur during a trial [16]. They also challenge researchers to consider inclusion and exclusion criteria of trials as well as the nature of risks identified during participant screening and the pre-consent process. While resentful demoralisation has been considered as a potential consequence of the randomisation process, detailed links between this and legitimate concerns that demoralisation may have significant mental health implications require further consideration. Furthermore, to date it is unknown to what level the risk of such mental health concerns is highlighted in participant information for physical rehabilitation trials, and such a review would be worthwhile. Indeed, this finding suggests that explicit reference to the potential psychological impact of being allocated to an inactive control could add to the risks identified in the participation information sheet. It might also be explored during the consenting process. Such measures may reduce recruitment and would therefore require sensitive application and a balance in judgement regarding how much this risk is raised with potential recruits. However, concerns with recruitment targets should not outweigh the transparency required for meeting the principles of informed consent.

A further issue identified in this study is the potential impact of compensatory behaviour change on trial outcomes. If the conclusions of trials are to be based on the changes in quantitative outcome measures, then it is apparent that an unmonitored active control may interfere with the capacity for any change to be evident or attributable to the intervention itself. The combination of narrative and concomitant reported change in physical outcomes for one participant in this pilot study in particular indicates that this may be the case. If so, recruitment to the trial itself can become an active intervention. While a study of the impact of participation in RCTs found no evidence of benefit or harm to RCT participants, compared to those involved in cohort (uncontrolled) studies of similar intervention [36], an impact of group allocation has been suggested previously [5], but has rarely been fully explored [4]. Such insights highlight the need to understand participants’ decision-making processes and action post randomisation, as well as the impact on behaviour of information given pre-consent. Indeed, McCambridge et al. ([5]:246) suggest that “fine grained attention to how participants react to what they are asked to do in research, and how this may impact on study outcomes, is needed” and our study supports that view. It also demonstrates the value of control group interviews and monitoring control group fidelity as part of process evaluation in order to understand and explain the trial results obtained. Such evaluation is not mentioned in reporting guidelines for pilot studies such as the Template for intervention description and replication (TIDieR), or the Consolidated standards of reporting trials (CONSORT) extension for pilot and feasibility studies. Funders and reviewers of pilot and feasibility trials may wish to pay closer consideration to these issues in future proposals.

In summary, this study has highlighted a number of areas that could impact on the ethical conduct of a rehabilitation trial and on the appropriate interpretation of outcomes. A number of suggestions have been made that may address these concerns and are summarised in Table 3. These will be considered in the development of the definitive ReTrain trial, but may also have relevance for other rehabilitation trials more broadly.

**Table 3:** Concerns and recommendations for informing the design and delivery of a definitive RCT

Concerns	Recommendations
Motivation for taking part	Recruiting staff to recognise that not all people are motivated by altruism
Endorsement by stakeholders (clinicians and funders)	Required for identifying potential participants and starting the recruitment process; training for Participant Identification Centre (PIC) clinicians to be aware of their “gate-keeping” role
Desperation for improvement	PIC clinicians and recruiting researchers to ensure potential participants are informed and understand the need for equipoise through careful reiteration of the current state of knowledge and potential risks as well as benefits of participation with the aim to facilitate appropriate decision-making
Abandoned to the control group	Consider more active forms of comparator, or waiting list control. Careful inclusion of interviews to ascertain group preference
Negative effects on mental health (if allocated to the control)	PIC clinicians and recruiting researchers to be aware of the vulnerability of potential participants; more active monitoring of adverse events in the control group, ensure mental health deterioration included in monitoring and reporting of adverse events
Compensatory behaviour change (control group participants seek their own version of the intervention)	Consider more active comparator or waiting list control; better monitoring of control group activity. Consider alternative trial designs such as rigorous pragmatic or observational cohort studies. Recommend process evaluations always include control group participants and update reporting check lists to this effect

### Limitations

The interviews were conducted at the end of the trial and therefore the experiences of allocation were retrospective. This, and the outcomes associated with participants’ allocation, may have impacted on the nature of the narratives. This is particularly the case with group allocation where initial thoughts are likely to have evolved over time and the subsequent experience of being in either the intervention or control group for several months. It is also important to acknowledge that these data were only from a sub-sample of the participants in the study. While the sampling frame target was achieved and a range of views was evident, it is possible that other insights were missed. Data saturation was not formally assessed in this study but there was sufficient repetition of ideas to suggest that key issues were captured.

The primary analysis was not completed by the interviewer. This has certain disadvantages as nuances in gesture and meaning may be lost. However, the inclusion of the interviewer in subsequent discussion of the analysis, as well as critical reflective discussions, negative case analysis and transparent analytical processes, helped ensure the rigour of the study and results presented.

## Conclusion

This paper has argued that participant effects within a rehabilitation pilot randomised controlled trial raise concerns about its ethical conduct and the interpretation of its results. It suggests that motivations to take part, and particularly the desperation for improvement in their condition, may create vulnerability in some study participants. This can hamper informed decision-making and result in undesirable impacts on members of the control group. Without adequate consideration of these effects, trials run the risk of undermining the health and wellbeing of some participants, and of drawing erroneous conclusions about group differences in effects.

We conclude that in the design and delivery of a future trial, increased attention should be accorded to these effects, and that process evaluation should give detailed consideration to the behaviours and experiences of participants in the control group. We further propose that such measures may help other rehabilitation trials to protect the wellbeing of participants and may enhance the credibility of their findings.

## Data Availability

All data generated or analysed during the current study are available from the Principle Investigator of the study Sarah G Dean on reasonable request.
